# Environmental sustainability and ruminant production: A UK veterinary perspective

**DOI:** 10.1002/vetr.4703

**Published:** 2024-09-26

**Authors:** Nick Britten, Sophie Mahendran

**Affiliations:** ^1^ Royal Veterinary College Hatfield UK; ^2^ Synergy Farm Health Rampisham Down UK

## Abstract

Environmental sustainability is an issue of growing importance within the livestock industry, particularly for farmed ruminants. Changes to farming practices made to improve sustainability can have an impact on the health and welfare of animals, and so become the concern of veterinary practitioners. This review outlines the metrics used to measure sustainability and how sustainability interacts with ruminant health and welfare, allowing practitioners to incorporate environmental considerations into their existing livestock work. Topics covered include nutrition, disease control, genetics and stocking density.

## INTRODUCTION

The general term ‘sustainability’ means to meet the needs of the present without compromising future generations’ abilities to meet their own needs.^1^ The UN has identified sustainable food consumption and production as a significant area of importance for achieving this, driving the need for development and employment of sustainable agricultural practices.^2^ This review of recent literature aims to outline the importance of sustainable ruminant production, how sustainability can be measured and how different aspects of ruminant production impact this sustainability. This should help increase awareness among UK veterinary practitioners of how their work impacts on sustainable agriculture practices.

## WHY DO WE NEED SUSTAINABILITY?

Environmental degradation is a critical issue, with scientists supporting the need for urgent action to manage the environment sustainably, particularly regarding ecological disruption, biodiversity loss and the ability of humans to produce food, energy and stable economies.^3^ Failure to address the degradation of the environment is predicted to lead to extreme climate events, and more closely connected to this review article, failure of food production.^4^ Environmentally conscious farming behaviour can be motivated by both altruistic and self‐interested reasons.^5^ The agricultural industry is under constant pressure to improve production, as food security is a national security issue, but it must take care to ensure that contemporary increases in productivity do not compromise future production; in other words, it must take care to be sustainable.^6^, ^7^

From the ‘self‐interested’ perspective, sustainability presents a commercial opportunity, with a meta‐analysis finding that consumers are willing to pay more for sustainably produced commodities.^8^ Participation in agri‐environmental schemes in the UK is on the rise, with 32,000 holdings signed up to the ‘Countryside Stewardship Scheme’ in 2023, a 94% increase from 2020.^9^ A survey of 20 UK dairy farmers participating in agri‐environmental schemes reported that only a minority had financial incentives as direct motivators of behavioural change, whereas more reported a desire to maintain a natural environment.^10^ A larger survey of UK farms identified natural systems, interpersonal relationships, policy and commercial factors as barriers to participation in more sustainable farming practices.^11^ A systematic review of factors affecting European farmers’ adoption of biodiversity‐friendly farming practices found that 108 different factors affected farmer decision making,^12^ demonstrating the breadth of factors influencing decision making on sustainable farming practices and highlighting the important role played by farm advisors, such as veterinary surgeons, in making such decisions.^12^

A conceptualisation of sustainability in the context of ruminant agriculture means meeting current production needs while not compromising future production and ensuring long‐term food security. This must include aspects of human and animal welfare, resource use, biodiversity preservation and the ma of waste.^13^ The UN sustainable development goals of eliminating hunger, availability of clean water, sustainable consumption and production, tackling climate change and preserving biodiversity and forests are all connected directly to ruminant agriculture.^2^ Animal health, human health and the environment are all intrinsically linked, a concept referred to as ‘One Health’.^14^ In this concept, veterinary surgeons have a professional responsibility for environmental protection,^14^ considering the sustainability implications of husbandry practices and the animal health and welfare implications of any changes made for sustainability reasons.

## INTERACTIONS BETWEEN SUSTAINABILITY AND WELFARE

The traditional definition of animal welfare revolves around the avoidance or prevention of negative states of experience for the animal,^15^ which is reflected in UK animal welfare legislation being based on the ‘five freedoms’.^16^ More recently, it has begun to incorporate consideration of the affective state of animals, and promotion of positive states has become a welfare aim.^17^, ^18^ UK law has now established a committee to evaluate the likely effects of government policy on the welfare of animals ‘as sentient beings’.^19^

While evaluations of welfare are often made subjectively, the use of a quantitative system of evaluation applied at different stages of a production system allows for an estimate of overall lifetime welfare via a life cycle assessment (LCA).^20^ This method allows integration of welfare as a metric into LCAs, which are frequently used when evaluating the sustainability of a farming system.^20^ Integrating welfare into evaluations of sustainability is important as changes to farming practices that improve production efficiency at the expense of welfare may be initially appealing but are not beneficial in the longer term.^21^ In a similar vein to One Health, human and animal welfare can be considered as linked under ‘One Welfare’, so improved animal welfare should also result in improved human welfare.^22^

As sustainability and welfare are both critical issues for the veterinary profession, the British Veterinary Association (BVA) has published a policy statement and action plan for sustainable animal agriculture.^23^ The BVA policy position highlights the need to consider animal health and welfare as sustainability goals in themselves and take care that animal health and welfare are not compromised for human need.^23^ Included in the BVA goals is the aim of encouraging consumers to purchase smaller amounts of animal product of higher welfare status—referred to as ‘less but better’—as an element of the ‘One Health’ responsibilities of the profession.^24^ With this framing, the role of the veterinary surgeon extends beyond the improvement of existing animal agriculture systems and into other interacting systems, a necessary development to achieve sustainability.

## CARBON FOOTPRINTING TO EVALUATE SUSTAINABILITY

Carbon footprinting is the most frequently used approach when evaluating the environmental sustainability of a farm. As mentioned above, the basic premise is to make a LCA of a product, for example, milk, to estimate the total emissions of greenhouse gases (GHGs) associated with its production. The GHGs are outlined in the Kyoto Protocol: carbon dioxide, methane, sulphur dioxide, perfluorocarbons, hydrofluorocarbons, sulphur hexafluoride and nitrogen trifluoride.^24^ The extent to which an evaluation of a product considers emissions is referred to as scope: scope 1 considers direct emissions only, scope 2 further includes emissions from energy used and scope 3 considers the impact of the entire supply chain.^25^ Scope 3 emissions make up over 90% of emissions for the agricultural sector.^26^ Scope 3 emissions are inherently difficult for producers to influence, as they are, by definition, not under their direct control, but despite this, there is widespread litigation regarding responsibility for scope 3 emissions and one ruling from the Netherlands (under appeal at the time of writing) has judged that a business can be held legally liable for their scope 3 emissions.^27^ LCA is used to estimate the total environmental impact of a product but can struggle to adequately quantify the interacting effects found in food‐producing systems.^28^ In addition, stakeholders report being concerned with soil, water, nitrogen, biodiversity and animal welfare, which are often not factored into estimates of GHG emissions.^20^, ^29^

The most frequently calculated metric in carbon footprinting is GWP_100_, which is intended to represent global warming potential over a timeline of 100 years and is typically represented as kilograms of carbon dioxide equivalent (Kg‐CO_2eq_), simplifying a set of distinct processes with similar effects into a single metric.^30^ Despite its appeal as a simplified single metric to represent an overall indication of general environmental sustainability, kg‐CO_2eq_ correlates poorly with other important metrics of sustainability, such as resource depletion or the release of toxic compounds.^30^ To facilitate comparison between livestock sectors that produce different products, the ‘emission intensity’ of a product is used to express how much emission is required per unit of production. For example, emission intensity can be expressed as kg‐CO_2eq_ per kg protein.^31^ However, using such metrics makes intensive operations appear more sustainable, owing to their high production, and may not adequately represent sustainability costs in terms of biodiversity, land degradation and animal welfare.^20^, ^32^

Estimates of global GHG production attributable to livestock farming are between 16.5% and 28%.^33^ In the dairy sector, enteric methane (which is different from methane from manure) is estimated to account for 47‒59% of all carbon emissions, and so the feed efficiency of cows is the main quantity evaluated by carbon footprinting.^34^ This might suggest that it makes carbon footprinting a better representation of cow efficiency rather than specifically of sustainability.^29^ A small population of high‐performing dairy cows is responsible for 45% of the world's milk production, but more than half of all dairy cows are in low‐intensity production areas.^35^ Even a country as small as the UK has intense regional variation in agricultural practices,^36^ which will necessitate a different approach to sustainability in those systems. Given the diversity of global farming systems, sustainability solutions that consider only the ecological elements of sustainability and not the wider social and economic implications are not appropriate in many circumstances, but little research considers all factors simultaneously.^37^

The large proportion of emissions attributable to methane also lends support to the use of a different model when evaluating the sustainability of livestock systems: GWP*.^38^ As methane is a much shorter‐lived GHG than CO_2_, the GWP* model allows the natural atmospheric removal of methane to be factored into both evaluation of the current situation and forward projections.^38^ Biogenic methane is a part of the natural carbon cycle and is a ‘flow’ form of carbon that transfers between states, compared with the ‘stock’ carbon released from fossil fuels when burned.^39^ This is better represented by the GWP* model, in which the rate of release of new methane is the main determining factor for warming potential.^38^ The comparison between GWP_100_ and GWP* illustrates an inherent weakness of using modelling to generate a metric: changing the model assumptions will change the apparent outcome despite evaluating an identical process.^40^, ^41^

There are a variety of tools available to farmers to evaluate the environmental impact of their farm. For farmers in the UK, the Agriculture and Horticulture Development Board provides a guide for choosing a carbon footprinting tool.^42^ The guidelines laid out by the Intergovernmental Panel on Climate Change split LCA methods into three tiers with increasing sophistication of the calculations.^40^ It has been suggested that tier 1 analysis is sufficient to detect a difference in GHG emissions between dairy farms of 8.7%.^41^ Several of these tools use tier 2–3 LCA methodology and offer outputs in GWP*, with price points starting at free.^42^ The following tools were evaluated for agreement by DEFRA, but there was little agreement found: Agrecalc (Agrecalc), Cool Farm Tool (Cool Farm Alliance), Carbon Footprint Tool (Eggbase), Farm Carbon Calculator (Farm Carbon Toolkit), Sandy (Trinity Agtech) and the Farm Carbon Calculator (SolAgro).^43^ Dairy UK, an organisation whose members account for 70% of milk processed in the UK, has set up a working group to attempt to improve the consistency of carbon footprint estimates.^44^ Despite the weaknesses of carbon footprinting, it still represents a simple, convenient and easily available way to estimate the environmental impact of livestock farming and is prevalent throughout the literature.

## GRAZING AND SUSTAINABILITY

More carbon is sequestered by grasslands than croplands because of organic matter decomposition and erosion at cultivation; thus, maintaining grasslands for the grazing of livestock may be beneficial to sustainability.^45^ While woodland sequesters more carbon than pasture, replacing pastures with woodland is not economically feasible.^46^, ^47^ Grassland primarily sequesters carbon in the soil, as opposed to woodland where carbon is largely stored in plant matter, which can make grassland a more resilient store than woodland given the risk of events such as drought or fires.^48^ When considering any change in land use for the purpose of carbon sequestration, it should be noted that the change itself may cause carbon release, as well as any compensatory land use changes elsewhere—there may ultimately be a net loss of soil carbon.^46^ The use of hedgerows as boundaries to grasslands can also increase the quantity of carbon sequestered.^49^

The greatest concentrations of soil carbon are sequestered in the first metre of soil, in either mineral‐associated or particulate forms.^50^ Approximately 60% of all carbon is sequestered at 30 cm or more.^51^ European soils become saturated at a concentration of approximately 50 g of carbon per kg of soil.^50^ Despite this limited capacity to sequester carbon, approximately 80% of European soils are not currently saturated.^50^ Increased fertiliser use, grazed stocking rate, cutting frequency and decreased plant diversity are significantly related to lower soil carbon.^51^

Rotational periods of intensive grazing followed by rest for the pasture have been shown to improve soil organic matter, chemical composition, microbiome and water retention when compared with continuous grazing at both low and high stocking densities.^52^ The precise effect of grazing on soil carbon sequestration depends on the temperature, water and nutrient availability to the pasture.^50^ The species grazing is important, with sheep typically causing more depletion of carbon sequestration than cattle.^50^ The practice of ‘adaptive multipaddock grazing’ in North America or ‘mob grazing’ in the UK has been shown to improve soil carbon sequestration, nitrogen stock, forage biomass and water retention.^52^, ^53^, ^54^ While environmentally beneficial, this approach to grazing management also alters foraging behaviour and has been reported to reduce animal growth.^55^

While grazing is not a guarantee of good welfare, grazed cows may have lower levels of lameness, mastitis and uterine disease.^56^ Consumers report that grazing is equally important to them as animal welfare, perceiving grazing as more ‘natural’ than housed systems.^57^ A rough calculation has suggested that it should be possible to maintain current yearly beef production in the United States entirely from grazing, suggesting that grassland has the capacity to play a greater role in beef production.^58^

## RUMINANT FEEDING AND SUSTAINABILITY

Ruminant enteric methane is primarily produced by the fermentation of cellulose in forages.^59^ Increasing proportions of dietary concentrates (thereby reducing the quantity of forages fed) could potentially reduce emission intensity by 15%.^59^ This solution must be pursued with caution as high (≥40%) proportions of dietary concentrates increase the incidence of digestive disorders and digital dermatitis, both of which will reduce the overall feed efficiency of the animal.^59^, ^60^ Direct manipulation of the rumen microbiome to reduce the quantity of methane produced has been trialled with many different compounds; nitrates, nitrites, fumarate, sulphate, chloroform and bromochloromethane all reduce methane production in the rumen but have significant toxic or environmental issues associated with them.^59^, ^60^, ^61^ Addition of homoacetogenic bacteria to an artificially simulated rumen has been demonstrated to reduce methane production but is yet to be trialled in vivo at the time of writing.^62^

There are some commercially available products that intervene in the rumen fermentation process. The most well established is the ionophore monensin, which increases the availability of reducing agents to form proprionate and reduces the number of protozoa in the rumen, with both processes generating less methane,^63^ although the methane reduction reported varies widely between studies.^64^ Health risks to humans of antimicrobial resistance caused by ionophore use in agriculture have been considered low, but recent evidence suggesting co‐selection for vancomycin resistance has led to calls for further investigation.^65^ Given the efficacy of monensin in reducing the prevalence of diseases that require the administration of antimicrobials,^66^ the costs and benefits of administration require careful analysis.

Seaweed (primarily red or brown) has been trialled extensively as an anti‐methanogenic feed additive for cattle and shown to significantly reduce methane emissions with no apparent production detriment.^67^ Red seaweeds are typically found in tropical environments and act on methanogenesis via bromoform, a compound that does not appear to accumulate in the tissues of the animal and has a very short half‐life, so it is considered of low risk to the environment if released.^68^ Brown seaweeds are found in temperate zones and thought to reduce methane emissions via phlorotannin compounds, but efficacy in studies is mixed.^69^ Blends of essential oils have also been trialled, and while the results are variable, a recent 6‐week trial has shown a time‐dependent effect of one blend, suggesting that longer‐term trials may be warranted.^70^ Another compound, 3‐nitrooxypropanol, directly inhibits an enzyme responsible for forming methane and has been demonstrated to reduce methane emissions in both beef and dairy cattle, although reports of effect sizes vary considerably between studies.^71^, ^72^

Protein sources for cattle feed are also a major opportunity for improved sustainability, particularly in finding alternatives to soy imported from South America.^73^ The use of locally grown crops, as opposed to imported soy, has been estimated to reduce global warming potential by up to 95% and land usage by up to 63%.^73^ Improving protein digestibility in feed may reduce nitrogen excretion and thus environmental contamination.^74^ In some cases, protein feeding can be reduced with no detriment to yield: an adequate (not excessive) supply of highly available protein is the goal of a diet.^75^

## RUMINANT FARMING AND WATER

Agriculture is a major usage of water worldwide, and the water footprint of products can be calculated using methods similar to those used for carbon footprinting.^76^ Beef and lamb have some of the highest water footprints of animal products, whereas milk is comparatively low.^76^ As with carbon footprinting, intensification of farming usually decreases water footprint per product made.^76^ Pollution of water sources with nitrogen and phosphorus by livestock farming is also an issue.^77^ One pathway to reduce phosphorus use in livestock is to reduce the rate of inclusion within diets for dry (non‐lactating) dairy cattle. Feeding restricted phosphorus to dry cows has been shown to improve calcium status around parturition when compared with a near identical diet with normal phosphorus with no apparent detrimental effects.^78^, ^79^, ^80^

## RUMINANT DISEASE CONTROL AND SUSTAINABILITY

Livestock diseases reduce production efficiency and increase the emission intensity of animal products.^81^ In cattle, estimates of the impact on GHG emissions have been calculated for specific disease events, including bovine viral diarrhoea (BVD), infectious bovine rhinotracheitis (IBR), Johne's disease, salmonellosis, liver fluke, mastitis and lameness^82^, ^83^, ^84^, ^85^, ^86^, ^87^ (see Table 1). In sheep, parasitic diseases have been the focus, specifically *Teladorsagia circumcincta*, rumen fluke and *Haemonchus contortus* infections.^88^, ^89^, ^90^, ^91^ While these studies provide some eye‐catching figures that can be used in discussions with producers, only one addresses the impacts of disease mitigation strategies.^82^ It is estimated by the Moredun Research Institute that UK livestock emissions could be reduced by 10% from improving health.^92^ The relative prevalence of a given disease and the ease of intervention determine the relative opportunity to address that disease.^93^ Mastitis, lameness, BVD, IBR and liver fluke are all significant diseases in the UK with high enough prevalence to give significant GHG impact if addressed,^87^, ^94^, ^95^, ^96^, ^97^ but in some circumstances, the proposed solution may cause as much environmental harm as the initial condition, and the lack of evidence examining the consequence of different mitigation strategies is a serious weakness of the existing literature.^81^

**TABLE 1:** Estimated increase in greenhouse gas (GHG) emissions per unit milk production of unmitigated disease across multiple dairy cow breeds

Disease	Estimated impact on GHG emissions	Reference
Liver fluke	10%	ADAS^82^
Johne's disease	25%	ADAS^82^
Bovine viral diarrhoea	20%	ADAS^82^
Salmonellosis	20%	ADAS^82^
Clinical mastitis	7%	Mostert et al^84^
Subclinical mastitis	3.7%	Gülzari et al^83^
White line disease	39%	Mostert et al^85^
Sole ulcer	33%	Mostert et al^85^
Digital dermatitis	4%	Mostert et al^85^

## RUMINANT MANAGEMENT AND SUSTAINABILITY

One of the perennial ideas surrounding sustainability is the concept of ‘dilution of maintenance’, where the resources required to keep an animal for a day are considered constant; thus, increased productivity represents a more efficient product.^81^ Improved reproductive efficiency and animal longevity also improve sustainability as fewer breeding animals are required to maintain production.^59^, ^61^, ^81^, ^98^ Optimising youngstock management is also of great importance, as surplus youngstock add to resource burden and any inefficiency in their rearing will also increase their lifetime carbon footprint.^99^, ^100^

The interaction between stocking densities and emissions is complex. Increasing stocking density in housed animals causes an increase in total carbon emissions from a system but typically results in a reduction in emission intensity.^61^ Increased stocking density at pasture may improve emission intensity but can also reduce the capacity of the soil to sequester carbon if overstocked.^51^, ^61^ Applying the principle of dilution of maintenance, stocking a system with the minimum number of maximally productive animals should be optimal for sustainability.^81^

## CATTLE GENETICS AND SUSTAINABILITY

Methane production has a genetic component in cattle, with the heritability of methane‐related traits estimated to be between 0.180 and 0.244.^101^ The genetics of both the host cow and the rumen microbiome have an impact on methane production, and although the effects do not appear to be correlated,^102^ the host genome appears to exert substantial influence on the rumen microbiome itself, which has the potential for 17% mitigation.^103^ Current genetic indexes require more data to be reliable, but there is significant potential to mitigate methane emissions from cattle directly by selective breeding.^104^

## SUMMARY

Sustainability is an increasingly important issue for the ruminant livestock sector and must go beyond just global warming potential to integrate animal health and welfare. For the veterinary surgeon, this will mean continuing to prioritise the health and welfare of livestock while giving increasing consideration to GHG, soil health and biodiversity implications of farming practices. There are a number of aspects of livestock production that typically involve the veterinary surgeon and interact with sustainability: feeding, housing, breeding and disease control. An enhanced awareness of these interactions as well as an understanding of how sustainability metrics are calculated will allow veterinary surgeons to provide the best advice on matters of sustainability. While research on many aspects of sustainability in livestock production is lacking, this is an area of intense research activity and is likely to continue to see substantial change in the near future.

## AUTHOR CONTRIBUTIONS

**Nick Britten**: Conceptulisation; funding aquisition; Writing—original draft preparation. **Sophie Mahendran**: Writing ‐ review and editing.

## CONFLICT OF INTEREST STATEMENT

The authors declare no conflicts of interest.

## ETHICS STATEMENTS

This article is a review of publically available published evidence. No ethical approval was required.

## Data Availability

This manuscript is a review. All sources referred to are publicly available.
